# TikTok and Eating Disorders: A Narrative Review

**DOI:** 10.1002/eat.70119

**Published:** 2026-05-02

**Authors:** Scott Griffiths, Sami Hondrogiannis, Noa Brittain

**Affiliations:** ^1^ Melbourne School of Psychological Sciences University of Melbourne Melbourne Australia

**Keywords:** algorithms, eating disorders, narrative review, review, social media, TikTok

## Abstract

**Objective:**

TikTok has rapidly become one of the world's most influential social media platforms, distinguished by its algorithmically personalized video feed. Its design prioritizes continuous, engagement‐optimized content delivery, raising questions about how such environments may shape eating disorder risk, symptom maintenance, and recovery. This narrative review synthesizes emerging research on TikTok and eating disorders.

**Method:**

We conducted a narrative review of studies identified through targeted database searches and reference screening. Findings were organized into four domains: (1) eating disorder‐relevant content on TikTok, (2) algorithmic systems structuring content distribution, (3) psychological and behavioral outcomes associated with content exposure, engagement, and creation, and (4) implications for clinical risk, recovery, and intervention.

**Results:**

Eating disorder‐relevant content on TikTok ranges from explicit pro‐eating disorder and pro‐recovery material to more socially normative dieting, fitness, and appearance‐focused content. Algorithm‐focused studies indicate that recommendation systems can disproportionately deliver such material to vulnerable users and may be more responsive to passive engagement signals (e.g., watch time) than to explicit actions (e.g., “liking”). Across study designs, greater exposure to and engagement with eating disorder‐related content appears to be associated with eating disorder symptoms and related phenomena. Personalized feeds may function as treatment barriers that warrant assessment in clinical settings, and psychoeducation about how recommendation systems operate may help clients recognize and disrupt harmful exposure cycles.

**Discussion:**

TikTok's algorithmically structured environment may interact with vulnerability to reinforce maladaptive comparison and behavioral cycles. Further longitudinal and clinically focused research is needed to clarify causal pathways and inform evidence‐based guidance for clinicians.

## 1 Introduction

TikTok reshaped social media by placing personalized content recommendations at the center of the user experience. Rather than organizing content around social connections, the platform delivers a continuous stream of algorithmically selected short‐form videos tailored to individual preferences. Videos recommended by the algorithm are designed to optimize engagement—meaning, to maximize the amount of time users spend on the platform. This recommendation‐first design, combined with a shift in the broader social media environment toward short‐form video, contributed to TikTok's rapid rise. However, the same features that optimize engagement also have implications for the development and course of eating disorders. In this narrative review, we examine TikTok's origins, user experience, and recommendation system, and synthesize the emerging empirical literature on TikTok and eating disorders.

## 2 Origins

TikTok emerged from the convergence of two short‐form video platforms developed in China in the mid‐2010s (Business of Apps 2025). Musical.ly, launched in Shanghai in 2014 by Alex Zhu and Luyu Yang, enabled users to create and share brief lip‐synced videos set to music and quickly gained traction among adolescents, reaching more than 80 million users, including a substantial audience in the United States. During the same period, ByteDance, founded by Zhang Yiming, was developing its own short‐form video platform in China. In 2016, it launched Douyin, a platform organized around algorithmic content recommendation rather than follower networks, allowing users to receive a personalized video stream upon opening the app. In 2017, ByteDance acquired Musical.ly, integrated its user base with Douyin's content recommendation algorithm, and relaunched the combined service internationally as TikTok in 2018. Today, Douyin operates exclusively within China with approximately 766 million users, while TikTok serves an international audience from its headquarters in Singapore. As of December 2025, TikTok has surpassed five billion downloads, reports approximately 1.6 billion active users worldwide, and has generated more than 23 billion dollars in revenue, with a user base that remains disproportionately young. Approximately 66% of its users are aged 18 to 35 years, with a gender split that slightly favors males (55.7%) (DataReportal 2025).

## 3 The User Experience

When someone downloads TikTok, they create an account and select a few broad interests such as “beauty”, “comedy”, or “fitness”, after which the app opens directly to a personalized home screen known as the “For You” page. Full‐screen videos begin playing immediately. Unlike earlier social media platforms, users do not need to follow friends or search for accounts before seeing content. For readers who grew up with platforms such as Facebook, this marks a significant shift. Facebook's 2008 Timeline displayed posts in reverse chronological order from friends, family, and followed pages, organizing content around existing social relationships. TikTok instead organizes content according to predicted user interest and engagement. It functions less as a social network and more as a recommendation‐driven media environment, delivering a tailored stream of short videos from the moment the app is opened. Each video can range from 15 s to 10 min (the mean video length is ~30 s), and an upward swipe brings the next video into view, creating a continuous stream with no endpoint, often described as an “endless scroll.” Although users can access other sections of the app, such as TikTok Live or a feed of followed accounts, these are secondary to the default For You page. In this way, TikTok sits at the forefront of a broader, 15‐year‐long trend in social media away from relationship‐based feeds and toward algorithmically curated attention streams (Chayka 2024).

## 4 The Algorithm

TikTok's defining feature is its content recommendation algorithm, which powers the For You page and determines the sequence of videos each user encounters. When the platform rose to prominence, many users described the uncanny experience of the algorithm appearing to match their humor, interests, and vulnerabilities, prompting refrains such as “my TikTok algorithm knows me better than I know myself” (Scanlon 2020). Recommendations are shaped by a wide array of signals including (i) explicit actions such as likes, comments, shares, follows, saves, direct messages, and content creation; (ii) subtler behavioral cues such as watch time, completion rates, replays, pauses, dwell time, swipe speed and direction, sound settings, profile visits, search queries, hashtag clicks, and repeated exposure to particular creators or themes; (iii) video‐level metadata such as captions, sounds, filters, and embedded text; and (iv) account and device‐level information including language, location, device type, and operating system. Because videos are brief and the feed effectively endless, the system receives dense, continuous feedback, enabling rapid refinement of predictions (TikTok 2024). Technically, this process relies on large‐scale machine learning, including deep learning systems that detect behavioral patterns and iteratively optimize for engagement. As these systems increase in complexity and repeatedly train on expanding datasets, their internal decision‐making processes become difficult to interpret, even for their designers, giving the algorithm “black box” properties in which outputs are observable but the pathways producing them remain opaque.

## 5 Existing Research on TikTok and Eating Disorders

We conducted a narrative review of studies identified through targeted database searches and reference screening. Our review is not intended to be exhaustive and instead focuses on our understanding of the extant research literature, including how best to organize it. Although still emerging, the literature on TikTok and eating disorders can be usefully organized into four interrelated domains: (1) eating disorder‐relevant content on TikTok; (2) the algorithmic systems structuring content distribution; (3) psychological and behavioral outcomes associated with content exposure, engagement, and creation; and (4) implications for clinical risk, recovery, and intervention. The domains overlap, and many studies span multiple areas, for example by linking algorithmic delivery patterns to individual symptoms.

### 5.1 Eating Disorder‐Relevant Content on TikTok

Qualitative content analyses are the most common method used to examine eating disorder‐related material on TikTok. In these studies, researchers systematically code samples of videos to identify recurring themes, narratives, and visual tropes, enabling analysis of how body ideals, dieting practices, and eating disorder‐related messages are portrayed. Collectively, this work suggests that many forms of content long considered harmful for individuals with body image concerns or eating disorders have migrated from earlier platforms such as Instagram and Facebook to TikTok. This includes content promoting highly restrictive diets, fitness and weight‐loss challenges that valorize thinness or leanness, subtle and overt reinforcement of narrow beauty standards, the moralization of food, and the stigmatization of fatness (e.g., Herrick et al. 2021; Dane and Bhatia 2023; Mancin et al. 2024; Minadeo and Pope 2022; Johanssen and Benzel 2025; Parnell et al. 2026). TikTok's visual, full‐screen format may heighten the salience of such material, as videos occupy the entire display and are encountered in rapid succession. Given the dynamic nature of social media, the existing corpus of harmful content will continue to evolve, sometimes emerging in new forms and at other times repackaging familiar narratives.

Qualitative interview studies complement this work by documenting how users describe the content they encounter. Individuals with lived experience of eating disorders report exposure to a broad spectrum of material, ranging from explicitly pro‐eating disorder content to subtler, socially sanctioned forms of body surveillance and dietary control (e.g., Parnell et al. 2026; Sjöström et al. 2024). Common examples include restrictive dieting advice, “what I eat in a day” videos, body transformation content, calorie‐counting practices, and aestheticized portrayals of thinness or muscularity framed as wellness or self‐improvement. Interviewees also describe how pro‐recovery content can coexist with, or slide into, material that romanticizes illness or intensifies comparison, blurring boundaries between helpful and harmful content. Rather than depicting TikTok as dominated solely by overt pro‐eating disorder communities, qualitative accounts emphasize the pervasiveness of aspirational, appearance‐focused content that feels culturally mainstream yet may heighten self‐surveillance and comparison among users vulnerable to eating disorders.

### 5.2 Algorithmic Systems Structuring Content Distribution

Research examining TikTok's algorithmic systems is inherently challenging because these systems are proprietary and function as black boxes. Importantly, algorithmic systems extend beyond recommendation engines to include moderation, filtering, and classification mechanisms that may suppress what certain users see. Few studies have meaningfully interrogated these systems through their design. Griffiths et al. (2024), using data donations from users, analyzed over 1 million videos and found that the recommendation algorithm disproportionately delivered appearance‐oriented, dieting, exercise, and toxic pro‐eating disorder content to users with eating disorders, independent of explicit “likes”. Similarly, Dondzilo et al. (2024) demonstrated that engagement with appearance‐related content predicted increased algorithmic recommendation of similar material, linking user behavior to downstream amplification. Taken together, these findings suggest that TikTok's algorithmic systems may differentially structure exposure in ways that reinforce vulnerability among users with eating disorders.

Computational research has further advanced this domain by examining platform‐scale dynamics using large datasets and machine learning methods. Zannettou et al. (2024) analyzed engagement patterns across large samples of TikTok videos to characterize how short‐form content is surfaced, interacted with, and amplified within recommendation‐driven feeds. Bickham et al. (2025) introduced the “EDTok” dataset, a systematically curated corpus of eating disorder‐related TikTok videos designed to enable longitudinal tracking of content dissemination, moderation practices, and thematic evolution over time. Chu et al. (2026) developed the “BigTokDetect” framework, a clinically informed multimodal machine learning model that integrates visual features, embedded text, audio signals, and behavioral metadata to detect pro‐bigorexia content that frequently bypasses keyword‐based moderation systems. By leveraging multimodal classification and large‐scale behavioral data, these studies provide tools for identifying harmful material, quantifying its spread, and evaluating the effectiveness of platform‐level moderation strategies.

Qualitative interview and focus group studies complement this work by describing how users experience these systems. Individuals with eating disorders, or those in recovery, portray the algorithm as an active force shaping both what feels available and difficult to avoid on the For You Page (e.g., Parnell et al. 2026; Sauerwein 2025; Sjöström et al. 2024). Adolescents recovering from anorexia nervosa described TikTok as having a “dual potential,” simultaneously fostering connection while exposing them to “triggering algorithms” and recurrent disordered content (Sjöström et al. 2024). Young people with eating disorder histories reported rapid escalation of exposure after minimal engagement, captured in themes such as “View One, See More,” “Morbid Curiosity,” and “From Helpful to Unhelpful,” where pro‐recovery content sometimes preceded more harmful material (Parnell et al. 2026). In an unpublished thesis by Sauerwein (2025), youth in recovery similarly emphasized the emotional harm associated with repeated exposure and the difficulty of escaping such content once it entered their feed. Collectively, these findings suggest that users develop intuitive “folk theories” of the algorithm as responsive and amplifying, experiencing its personalization as both supportive and capable of intensifying exposure to triggering material.

### 5.3 Psychological and Behavioral Outcomes Associated With Content Exposure, Engagement, and Creation

Most studies in this domain are cross‐sectional and report associations between exposure to and engagement with eating disorder‐relevant TikTok content and poorer body image, greater internalization of beauty ideals, and elevated eating disorder symptoms (e.g., Dane and Bhatia 2023; Mink and Szymanski 2022; O'Connor et al. 2024). For example, Dondzilo et al. (2024) found that engagement with appearance‐ and eating‐related content predicted increased upward appearance comparisons and higher eating disorder symptomatology, suggesting that interaction with such material may intensify its psychological impact rather than merely reflect pre‐existing concerns. Caravelli et al. (2025) found associations between the use of TikTok appearance‐improving facial filters and body dissatisfaction, extending the existing exposure‐ and engagement‐based literature to content creation.

Longitudinal, experimental, and clinically sampled evidence remains limited. Although broader social media research has produced mixed findings regarding change over time (e.g., de Valle et al. 2021; Goh et al. 2025), relatively few studies have prospectively examined TikTok in relation to eating disorders, and those that have contain methodological limitations that complicate interpretation. Maes and Vandenbosch (2022) studied 229 Belgian adolescent girls across three waves (~9 months) using random‐intercept cross‐lagged panel models, an analytic approach that appropriately separates within‐person change from between‐person differences. They found no significant cross‐lagged effects between self‐reported TikTok use and body image; however, the study was highly underpowered to detect within‐person effects. Strickland et al. (2025), in contrast, followed 252 female undergraduates over 9 weeks and reported that higher baseline disordered eating predicted greater subsequent engagement with restrictive TikTok content, with both increasing over time. However, their analytic approach did not disaggregate within‐ and between‐person variance, making it unclear whether the observed effects reflect true within‐person change. Experimental studies further indicate short‐term causal effects insofar as young women exposed to algorithmically recommended pro‐anorexia, appearance‐focused, or dieting‐related content report reductions in body satisfaction and self‐esteem, alongside increased thin‐ideal internalization (e.g., Blackburn and Hogg 2024; Davis et al. 2025; Pryde and Prichard 2022). Pruccoli et al. (2022), in a study of 78 children and adolescents with eating disorders, found TikTok was the most frequently used social media platform, with 59% reporting that TikTok reduced their self‐esteem. The children and adolescents who reported reduced self‐esteem were significantly more likely to have searched and browsed dieting and eating disorder recovery content. Nevertheless, given the scarcity of longitudinal and clinical research, it remains unclear whether sustained TikTok use independently contributes to worsening eating disorder symptoms over time. Experimental and cross‐sectional work has pointed toward familiar mechanisms. For example, Pryde and Prichard (2022) found evidence that state appearance comparisons mediate the effect of exposure to TikTok fitspiration videos and body image, and Griffiths et al. (2024) found that folks with eating disorders reported more making more frequent TikTok‐specific appearance comparisons than healthy controls (Cohen's *d* = 0.86—a large effect size). Overall, current evidence points to meaningful short‐term psychological effects and behavioral reinforcement processes, while longer‐term trajectories require further study.

### 5.4 Implications for Clinical Risk, Recovery, and Intervention

This fourth domain is important because the clinical implications of TikTok arise from the interaction of content, algorithmic content distribution, and the psychological correlates of content exposure and engagement. If eating disorder‐relevant content is prevalent and algorithmic systems shape who sees it, these dynamics may influence treatment trajectories and relapse risk. For example, emerging evidence indicates that whilst individuals with eating disorders are *slightly* more likely to actively engage with dieting‐focused content, they are *much* more likely to be exposed to this content in the first place, suggesting the presence of self‐reinforcing feedback loops based on passive signals of engagement (e.g., watch time) rather than active signals (“liking”) (Griffiths et al. 2024). This might help explain qualitative accounts wherein individuals in recovery report being aware of algorithmic curation yet nonetheless struggle to avoid triggering material once it permeates their personalized feed (Sjöström et al. 2024). Griffiths et al. (2024) further reported that individuals with eating disorders found it substantially more difficult to disengage from TikTok compared to healthy controls despite their objective data revealing a statistically identical amount of use. This implies that it is not social media use in general that is driving eating disorder symptoms, but something at the intersection of content, algorithmic content curation, and pre‐existing vulnerabilities (e.g., a tendency to engage in appearance comparisons). Clinicians may therefore need to assess social media environments explicitly, explore engagement patterns, and incorporate strategies for managing algorithmically curated exposure into relapse prevention. However, the empirical base in clinical populations remains limited, and longitudinal evidence is sparse, leaving open questions about long‐term symptom trajectories.

Clinical guidance remains underdeveloped, but several pragmatic considerations follow from the current evidence. Clinicians might screen for frequent TikTok use and evaluate whether a client's For You Page functions as a treatment barrier, rather than assuming that use itself is inherently problematic. Simply refraining from “liking” harmful content is unlikely to shift recommendations, given the algorithm's sensitivity to passive signals such as watch time. More explicit actions, including using the “Not Interested” feature, blocking creators, reporting hashtags, or refreshing the For You feed, may help disrupt harmful cycles, though these strategies have not yet been empirically validated and algorithmic biases may re‐emerge over time. At the same time, it may be useful to move beyond framing TikTok as uniformly harmful. The platform can expose users to both recovery‐oriented and symptom‐relevant content, often within the same feed, suggesting that its effects depend on how it is encountered and engaged with. Helping clients understand how algorithmic systems amplify attention patterns, and how these may interact with eating disorder vulnerabilities, aligns with emerging intervention research suggesting that brief social media‐focused strategies can reduce eating disorder risk and improve body image among frequent users (e.g., Ladwig et al. 2025).

## 6 Future Directions for Research

Future research should prioritize designs that can better isolate causal pathways between TikTok and eating disorder onset, maintenance, and recovery. There is a particular need for adequately powered longitudinal studies that disaggregate within‐person change from between‐person differences, alongside experimental paradigms that more closely approximate real‐world, algorithmically curated exposure rather than static or researcher‐selected stimuli. Progress in this area will depend on improved access to ecologically valid behavioral data, and data donation approaches, in which participants securely share their own platform data. Complementing this, large‐scale metadata scraping and computational auditing of publicly available TikTok content can enable systematic tracking of how eating disorder‐relevant material is produced, circulated, moderated, and transformed over time. Integrating these approaches with clinical cohorts will be especially important. More broadly, interdisciplinary collaboration across clinical science and computational social science will be useful in the shift toward mechanistic models that can inform intervention, policy, and platform design.

## 7 Conclusion

TikTok exemplifies a broader transformation in social media toward algorithmically curated, engagement‐optimized environments that actively shape patterns of exposure. Across domains, the emerging evidence suggests that eating disorder‐relevant content is prevalent on the platform, that recommendation systems might preferentially expose vulnerable users to this content, and that exposure and engagement with this content are associated with adverse psychological outcomes. Although the literature remains relatively young and longitudinal data are limited, findings from content analyses, algorithm‐focused studies, experiments, and qualitative interviews converge on a common theme: TikTok's architecture may interact with pre‐existing vulnerabilities to intensify comparison, reinforce restrictive behaviors, and embed symptom‐relevant cues within everyday digital life. At the same time, the platform's design does not operate in isolation from user agency, cultural norms, or broader media ecosystems. Rather than framing TikTok as uniquely harmful, it may be more accurate to view it as an influential case study in how algorithmically structured attention environments can shape eating disorder risk and recovery trajectories. Rigorous longitudinal, clinical, and interdisciplinary research will be essential to clarify these dynamics and inform proportionate responses.

## Author Contributions

**Scott Griffiths:** conceptualization, supervision, writing – original draft preparation, writing – review and editing. **Sami Hondrogiannis:** data curation, writing – original draft preparation, writing – review and editing. **Noa Brittain:** data curation, methodology, writing – original draft preparation, writing – review and editing.

## Lived Experience Involvement Statement

No specific efforts were undertaken to involve persons with lived experience in the study design or execution, or in the preparation of this manuscript.

## Funding

Scott Griffiths receives funding from the National Health and Medical Research Council (grant numbers: 1179321).

## Ethics Statement

The authors have nothing to report.

## Conflicts of Interest

Scott Griffiths has provided unpaid expertise to government bodies in Australia, Singapore, and the United Kingdom; unpaid expertise to Meta; paid expertise to TikTok; and a combination of paid and unpaid expertise to education‐ and mental health‐related organizations in Australia. The authors affirm that no external parties or organizations had input into the design, conduct, analysis, or interpretation of this narrative review.

## Data Availability

Data sharing not applicable to this article as no datasets were generated or analysed during the current study.
